# Microvascular Free-Flap Outcomes at a Low-Volume Canadian Center: A Retrospective Analysis

**DOI:** 10.7759/cureus.115021

**Published:** 2026-08-23

**Authors:** Kurtis D Kerr, Teri-Lynn Parrell, Lisa Hussey, Douglas Angel, Jamie Tibbo

**Affiliations:** 1 Surgery/Otolaryngology, Memorial University of Newfoundland, St. John's, CAN; 2 Otolaryngology, Newfoundland and Labrador Health Services, St. John's, CAN

**Keywords:** fellowship training, flap salvage, head and neck reconstruction, low-volume center, microvascular free flap, operative takeback, surgical outcomes

## Abstract

Introduction: This descriptive outcomes study evaluated microvascular free-flap outcomes at a low-volume Canadian center where procedures were performed by surgeons with high-volume fellowship training.

Methods: A retrospective chart review included 65 consecutive patients who underwent a microvascular free-flap surgical procedure from 2016 to 2021 in St. John’s, Newfoundland and Labrador. Six annual institutional failure rates were compared with six published high-volume-center failure rates using Welch’s independent-samples t-test. The primary outcome was total or partial free-flap failure within 14 postoperative days. Secondary outcomes included postoperative complications, operative takeback, and flap salvage.

Results: The mean age of the study sample was 63.29 years; 41 were male, and 24 were female. The radial forearm was the most common harvest site (n = 32, 49%). Post-operative complications occurred in 24/65 patients (37%). Eight patients underwent flap-related operative takeback (8/65, 12%); seven required surgical salvage, corresponding to a partial-failure rate of 7/65 (11%), while one underwent exploration without intervention. All seven flaps requiring intervention were successfully salvaged, yielding a salvage rate of 7/7 (100%) and an overall flap success rate of 65/65 (100%). The mean annual institutional failure rate was significantly lower than the mean rate reported across the six high-volume comparator studies (0% versus 3.33%, p = 0.003).

Conclusion: Favourable free-flap outcomes and complete salvage of threatened flaps were achieved at this low-volume regional center. These findings suggest that successful microvascular reconstruction can be maintained within an experienced and appropriately supported low-volume program staffed by surgeons with high-volume fellowship training.

## Introduction

Microsurgical free-flap transfer within otolaryngology-head and neck surgery represents a key advancement in reconstructive management following oncologic resection [1]. Despite its transformative role, the technical complexity and potential for flap failure have placed pressure on surgeons to maintain high annual case volumes [1,2]. The prevailing rationale for this emphasis is that, given the intricate nature of microvascular surgery, consistent repetition is required to preserve technical proficiency and minimize the risk of failure [1-3].

Failure rates reported in the literature reflect outcomes across both high- and low-volume surgical centers. In high-volume centers, outcomes are consistently favorable. Crawley et al. identified a complete failure rate of 4.8% among 892 free-flap patients over 10 years, with mean postoperative salvage at 3.64 days and mean failure at 8.44 days [2]. Similarly, Corbitt et al. reviewed 3,090 patients across 28 surgical interventions and reported a 1.3% failure rate, yielding an overall success rate of 96.4% when revision cases were included [3]. A separate high-volume study involving 1,530 breast, head, neck, and extremity reconstructions demonstrated a 96% success rate, with 4.6% of participants requiring additional medical or surgical management for partial flap loss [4]. Taken together, these data indicate that free-flap failure rates in high-volume centers remain low, typically below 5%.

By contrast, outcomes from low-volume centers have been more variable. Verhelst et al. analyzed 129 patients over 20 years in a low-volume Belgian center and reported a 12.4% failure rate, which the authors attributed to stringent definitions of flap loss rather than true clinical outcomes [5]. In another review of 136 patients over 20 years, Klosterman et al. found a 96% success rate, though 16% of patients required revision procedures; among these, a 60% salvage rate was achieved, underscoring the influence of surgical experience on outcomes [6]. Similarly, Brennan et al. reported a 96% initial success rate in 27 patients treated over three years, with no complete flap failures [7]. Collectively, these findings suggest that while institutional case volume may influence outcomes, other mediating factors - particularly surgeon experience and technical proficiency - play critical roles in flap success.

Multiple preoperative, intraoperative, and postoperative variables have been associated with increased risk of free-flap failure. Alcohol abuse is a significant predictor of complications, with postoperative withdrawal increasing the risk of flap failure 3.7-fold [2,8,9]. Smoking history similarly raises the odds of failure by 3.4 times [9]. Pulmonary comorbidities have been linked to partial flap loss, while severe systemic disease (American Society of Anesthesiologists (ASA) IV classification) increases postoperative complication risk, though not always significantly [8]. Prior treatments such as radiotherapy and previous flap attempts have also been implicated in postoperative infection and flap loss [10,11].

Intraoperative factors further influence outcomes. Immediate pedicle revision and anastomosis to the lingual vein significantly increase the probability of failure [2,4]. Prolonged operative time, greater intraoperative blood loss, and extended anesthesia have each been associated with higher complication rates [9,12]. Importantly, flap ischemia duration is a key determinant of viability: for every additional five minutes of ischemia, the likelihood of failure increases by approximately 6% [2,13].

Postoperative factors such as venous thrombosis - accounting for 37.5-60% of flap failures, typically within the first three days - remain major causes of flap loss [5,6]. Wound dehiscence, infection, and skin breakdown also contribute significantly to postoperative complications [3,5,6]. Although these perioperative and intraoperative factors are important, surgical expertise remains central to successful outcomes. Corbitt et al. identified that 12.5% of failures were attributable to technical issues during flap harvest, underscoring the influence of operator skill [3]. Other studies have shown that outcomes improve with accumulated experience, emphasizing the long-term benefits of early, high-volume surgical exposure [6,11,13].

These findings suggest that high-volume fellowship training may mitigate the limitations of low institutional case volumes by maintaining advanced microsurgical proficiency and decision-making skills.

The primary aim of the present study was to descriptively evaluate microvascular free-flap outcomes at a low-volume surgical center where procedures were performed by surgeons with high-volume fellowship training. A secondary aim was to characterize preoperative comorbidities, intraoperative factors, and postoperative complications within the cohort. The study was originally conceived as a case-control analysis; however, because no total flap failures occurred, formal comparison of failure and success groups and formal risk-factor analysis were not possible. The study was therefore conducted as a descriptive outcomes analysis, with institutional results contextualized against published high-volume-center failure rates. We hypothesized that free-flap outcomes at our institution would compare favorably with those reported by higher-volume centers.

## Materials and methods

This study received ethical approval from the Health Research Ethics Board of Memorial University (Ref # 2022.201). Patient identifiers, including names, dates of birth, healthcare numbers, and specific dates of interventions and hospital admissions, were removed during data collection to ensure anonymity. The study was originally designed as a case-control analysis comparing flap failures with successful outcomes. After data collection, no total flap failures were identified, precluding formation of a failure group and formal risk-factor analysis. The study was therefore reclassified as a descriptive outcomes analysis. This descriptive analysis was completed on patients who underwent a microvascular free-flap surgical procedure by two head and neck surgeons practicing at St. Clare’s Mercy Hospital in St. John’s, Newfoundland and Labrador. The sample for the study was identified by a review of all free-flap procedures performed from 2016 to 2021 by these surgeons, both of whom had completed high-volume microvascular fellowships. A failed free-flap procedure was defined as the total loss of flap viability within 14 days of the postoperative period. A partial free-flap failure was defined as a flap requiring surgical intervention to maintain viability within 14 postoperative days [2-4,11]. An operative takeback was defined as any patient requiring anatomical evaluation in the operating room (OR), whereas a salvage was defined as preservation of flap viability following operative takeback and any required surgical intervention. Primary success was defined as flap survival without direct surgical intervention. Overall success included both primary successes and successfully salvaged flaps. Included free flap types were radial forearm, fibula, anterolateral thigh, and posterior tibial.

Postoperative free-flap monitoring followed an established institutional protocol. Patients initially received ICU-level postoperative care with frequent clinical assessment and Doppler monitoring of flap perfusion, followed by a gradual step-down in monitoring intensity as the postoperative course remained stable. Decisions regarding operative takeback were made by the treating otolaryngology-head and neck surgery team when clinical or Doppler findings raised concern for flap compromise.

The two surgeons involved in this investigation both completed five-year surgical residencies in Otolaryngology-Head and Neck Surgery, which are accredited by the Royal College of Physicians and Surgeons of Canada. Both surgeons completed high-volume surgical fellowships in microvascular surgical reconstruction that were one year in length. Each surgeon performed approximately 100 microvascular free flaps, including radial forearm, anterolateral thigh, fibular, and posterior tibial free flaps in their respective fellowships. One surgeon has been practicing full-time in this area since 2010, while the other has been practicing full-time in this area since 2015 at St. Clare’s Mercy Hospital in St. John’s, Newfoundland and Labrador. As above, all microvascular free-flap surgeries identified in the 2016-2021 study period were performed by these two surgeons.

Study sample and outcome measures

A census sampling approach was used. All consecutive patients who underwent microvascular free-flap reconstruction by the two participating surgeons at St. Clare’s Mercy Hospital between 2016 and 2021 were considered for inclusion. No a priori sample-size calculation was performed because all eligible cases during the predefined study period were included. Patients were included if they underwent head and neck microvascular free-flap reconstruction during the study period and sufficient documentation was available to determine the primary outcome. Records were excluded if the procedure occurred outside the study period, was not a microvascular free-flap reconstruction, or lacked sufficient documentation to determine flap outcome. Eligible cases were identified through the regional health authority’s OR database, yielding 65 patient records; no identified eligible cases were excluded. Corresponding electronic health records were then reviewed using a predefined set of study variables. Demographic, comorbidity, operative, and postoperative outcome data were abstracted consistently for each case.

The primary outcome measure was the rate of partial or total free-flap failure among these 65 patients. Secondary outcome measures included preoperative comorbidities, intraoperative characteristics, and postoperative complications that were prespecified based on factors reported in the literature and were systematically abstracted from the medical record to characterize the cohort [3-6,8-13].

Identified patients’ electronic health records were screened for preoperative risk factors associated with surgical readiness and free-flap failure. Identified risk factors that were supported in the literature included hypertension, diabetes, ischemic heart disease, dyslipidemia, peripheral vascular disease, history of thrombosis, smoking, drug use, alcohol use, prior chemotherapy, history of head and neck surgery, and/or history of head and neck radiation [2,8-11]. From the electronic health records, intraoperative risk factors were identified through the same process and included estimated blood loss and free-flap ischemia time, along with postoperative complications that were associated with surgical takeback, which included venous thrombosis, hematoma evacuation, removal of adhesions, and Doppler wire replacement [2,3,5,6,9,12,13]. Post-operative complications not associated with OR takeback or free-flap failure were also identified. While these secondary outcome measures were not subjected to formal statistical analysis, they were used to characterize the patient sample. When a variable was not documented in the medical record, it was treated as missing and was not imputed.

Comparator studies were selected from published high-volume microvascular free-flap series that reported an explicit flap-failure rate and used outcome definitions sufficiently similar to permit contextual comparison with the present cohort. Since the comparator studies differed in patient populations, reconstructive sites, follow-up periods, and failure definitions, the comparison was intended to provide contextual benchmarking rather than establish comparative effectiveness.

Statistical analysis

Statistical analyses were performed using IBM SPSS Statistics for Windows, Version 30 (Released 2024; IBM Corp., Armonk, New York, United States). The analysis compared six individual failure rates reported by selected high-volume centers [2-4,11,13,14] with six annual institutional failure rates from 2016 through 2021. For each calendar year from 2016 through 2021, the institutional failure rate was calculated as the number of total flap failures divided by the number of free-flap procedures performed that year. The six published comparator rates had a mean of 3.33% (SD 1.50%), whereas all six annual institutional failure rates were 0%, resulting in a mean and SD of 0%. Levene’s test indicated unequal variances, F(1,10) = 27.26, p < 0.001; therefore, Welch’s independent-samples t-test was used. Statistical significance was set at p < 0.05. Secondary outcome measures were summarized using descriptive statistics and were not subjected to formal inferential analysis. Comorbidities were highlighted to better characterize the study sample.

## Results

**Figure 1:**
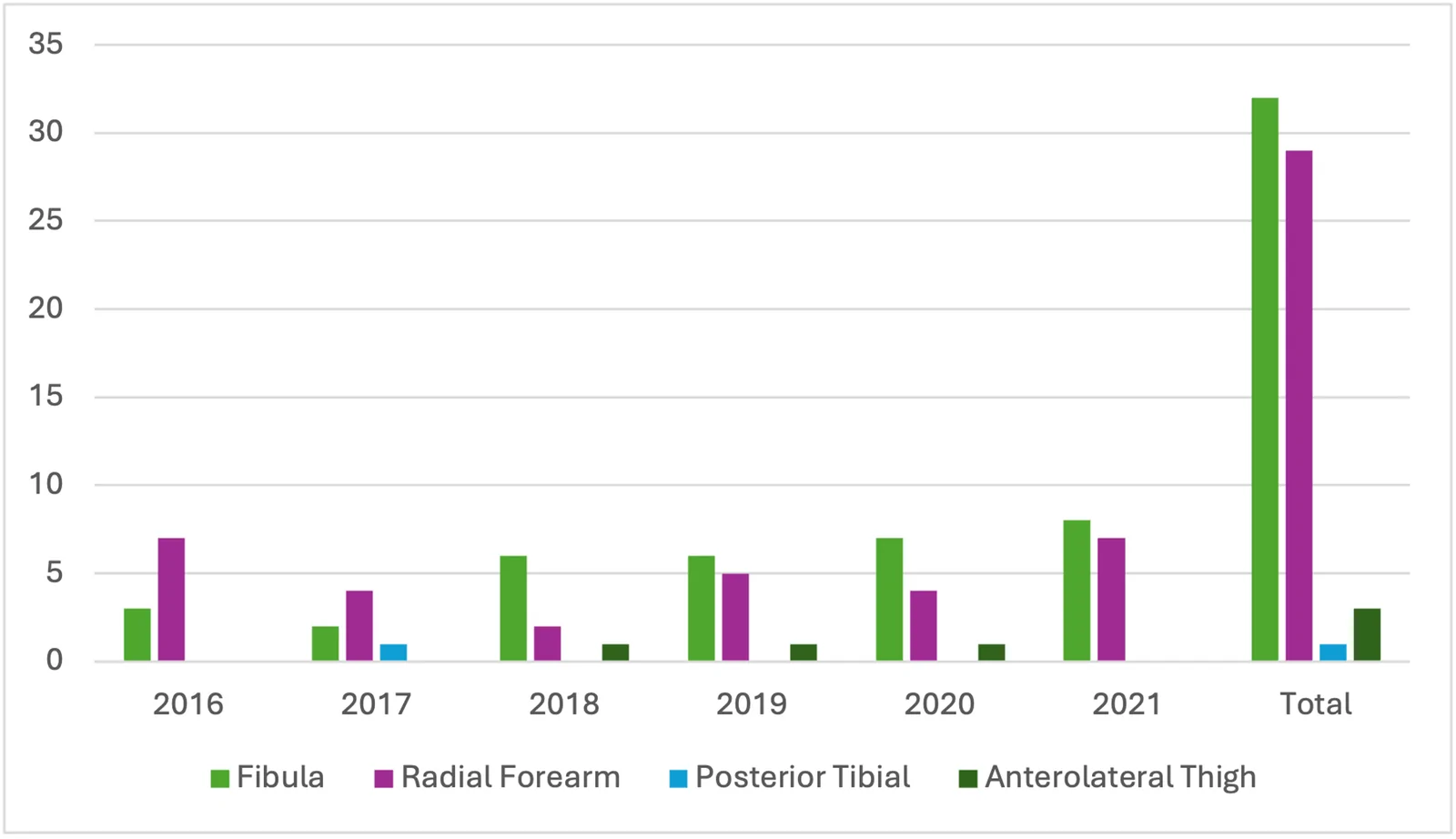
Distribution of Microvascular Free-Flap Harvest Sites Distribution of flap types among the 65 microvascular free-flap procedures included in the study. Radial forearm free flaps were the most common, followed by fibula, anterolateral thigh, and posterior tibial free flaps. The figure was created by the authors using Microsoft Excel (Microsoft Corp., Redmond, WA, USA).

**Figure 2:**
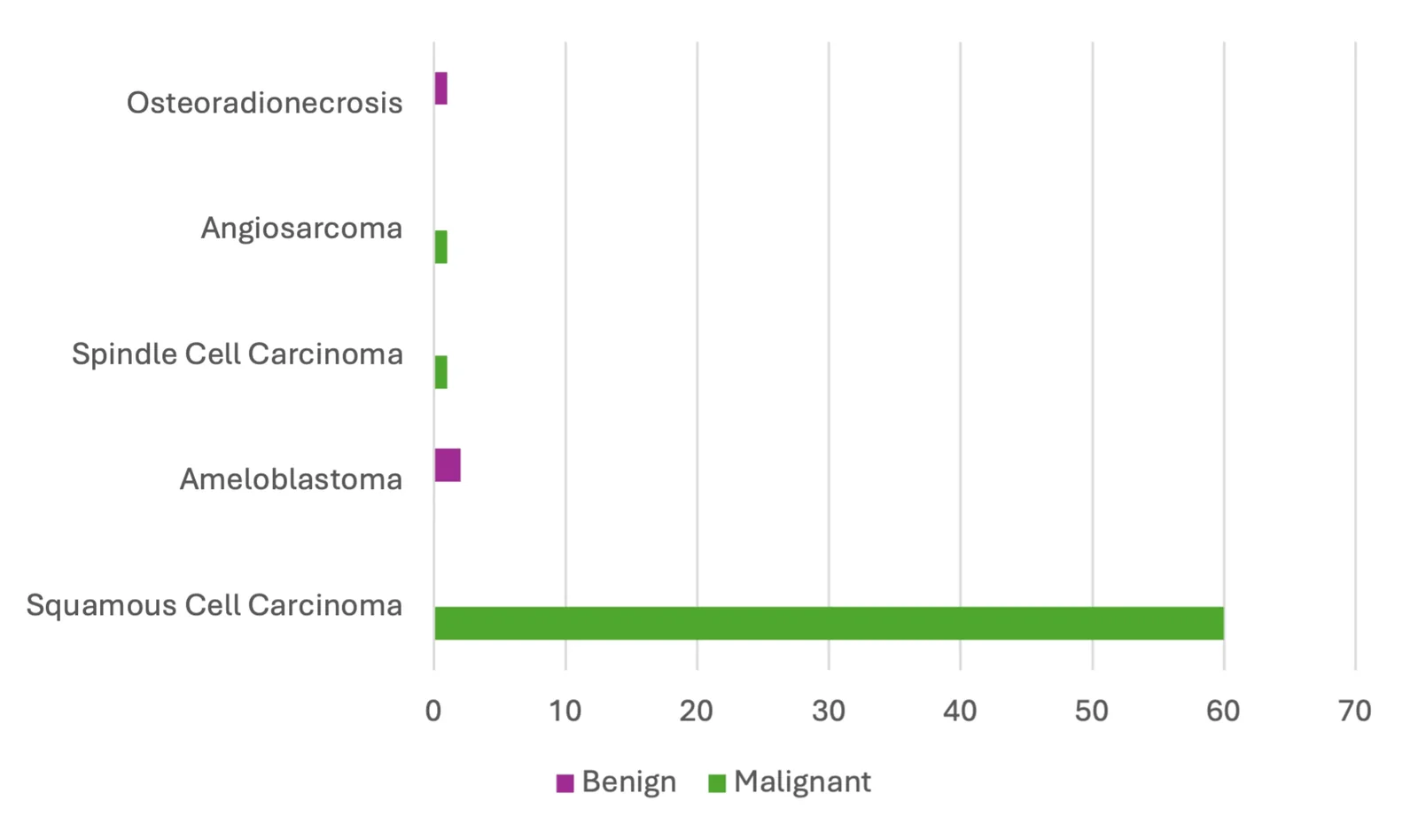
Distribution of Presurgical Diagnoses Distribution of presurgical diagnoses among the 65 patients included in the study. Squamous cell carcinoma was the most common diagnosis, accounting for 60/65 cases (92%), followed by ameloblastoma in 2/65 cases (3%), and spindle cell carcinoma, angiosarcoma, and osteoradionecrosis in 1/65 case each (2% each). The figure was created by the authors using Microsoft Excel (Microsoft Corp., Redmond, WA, USA).

Of the 65 patients identified for study analysis, 41/65 patients (63%) were male, and 24/65 (37%) were female (mean (SD) age was 63.2 (11.2) years). The three most common comorbidities were hypertension (36/65, 55%), history of smoking within three months prior to surgery (31/65, 48%), and dyslipidemia (27/65, 42%) (Table 1). Of all free-flap surgeries completed, the radial forearm was the most common harvest site (32/65, 49%), followed by the fibula (29/65, 45%), anterolateral thigh (3/65, 5%), and the posterior tibia (1/65, 2%) (Figure 1). Squamous cell carcinoma was identified as the most common presurgical diagnosis in 60/65 (92%) patients; two patients were diagnosed with ameloblastoma (2/65, 3%), and one patient each with spindle cell carcinoma (1/65, 2%), angiosarcoma (1/65, 2%), and osteoradionecrosis (1/65, 2%) (Figure 2).

**Table 1:** Patient Demographics, Comorbidities, and Clinical Characteristics Data are presented as n (%) unless otherwise specified. Percentages are calculated using the total study cohort of 65 patients as the denominator.

Total Free Flaps (N=65)	n	(%)
Gender		
Male	41	63
Female	24	37
Comorbidity		
Chronic Obstructive Pulmonary Disease	9	14
Emphysema	1	2
Obstructive Sleep Apnea	8	12
Hypertension	36	55
Type 2 Diabetes Mellitus	19	29
Ischemic Heart Disease	11	17
Dyslipidemia	27	42
Atrial Fibrillation	5	8
History of Pulmonary Embolism	2	3
Immunodeficient	3	5
Peripheral Vascular Disease	5	8
Chronic Kidney Disease	2	3
Acute Myeloid Leukemia	1	2
Hepatitis C	1	2
Hemochromatosis	1	2
Osteoradionecrosis	1	2
Prior Chemotherapy and Radiation (Non-head and Neck Cancer)	6	9
History of Smoking Within Three Months of Surgery	31	48
History of Alcohol Use	21	32
Previous Head and Neck Surgery	18	28
Prior Drug Use	3	5
Previous Head and Neck Radiation	7	11

The mean failure rate across the six selected high-volume centers was 3.33% (SD 1.50%), compared with 0% (SD 0%) across the six annual institutional observations [2-4,11,13,14]. Welch’s t-test demonstrated a statistically significant difference, t(5) = 5.46, p = 0.003, with a mean difference of 3.33 percentage points and a 95% confidence interval of 1.76 to 4.90 percentage points (Table 2).

**Table 2:** Comparison of Free-Flap Failure Rates Between the Present Study and Selected High-Volume Studies Values are presented as n, n/N, or percentage. Six published study-level failure rates were compared with six annual institutional failure rates from 2016 through 2021 using Welch’s independent-samples t-test. The mean comparator failure rate was 3.33% (SD 1.50%), compared with an institutional annual mean of 0% (SD 0%). Welch’s t-test demonstrated a statistically significant difference, t(5) = 5.46, p = 0.003.

Study	Sample Size (n)	Flap Failures (n/N)	Failure Rate (%)
Crawley et al. [2]	892	43/892	4.8
Corbitt et al. [3]	3090	40/3090	1.3
Las et al. [4]	1530	67/1530	4.4
Mucke et al. [11]	451	18/451	4.0
Formeister et al. [13]	361	6/361	1.6
Kudpaje et al. [14]	1001	40/1001	3.9
Present Study	65	0/65	0.0

Across all 65 free-flap surgeries, mean estimated blood loss was 568.75 mL, mean ischemia time was 149.7 minutes, mean hospital stay was 18.1 days, and eight patients underwent flap-related operative takeback (8/65, 12%). Seven required surgical intervention (7/65, 11%), while one underwent exploration without intervention (1/65, 2%). Seven patients met the definition of partial flap failure as operative intervention was required to maintain flap viability. One patient (2%) had a neurosurgical complication requiring operative management (cerebrospinal fluid leak), while another needed neurosurgical exploration without intervention (2%, as above). These two patients required neurosurgical re-intervention for complications related to the neurosurgical component of their care; neither event involved the otolaryngology team or the free flap.

Post-operative complications occurred in 24/65 patients under the otolaryngology service (37%): post-operative infection (5/65, 8%), fistula development (3/65, 5%), delirium (2/65, 3%), aspiration pneumonia (1/65, 2%), cerebrospinal fluid leak (1/65, 2%), cerebrovascular accident (1/65, 2%), hypernatremia (1/65, 2%), minimal posterior pedicle separation (1/65, 2%), pedicle breakdown (1/65, 2%), and pressure sore development (1/65, 2%), orocutaneous fistula (1/65, 2%), post-operative oozing (1/65, 2%), gastrostomy tube (G-tube) insertion for dysphagia (2/65, 3%), significant post-operative nausea and vomiting (1/65, 2%) and physical decompensation (1/65, 2%). One patient (1/65, 2%) who underwent a radial forearm free-flap died subsequent to a bowel perforation in the intensive care unit while under general surgery care. This was identified as a secondary complication from gastrostomy tube placement. This was not related to care or intervention provided by the head and neck surgery department; the flap remained viable, and the death was not classified as a flap failure. Another patient (1/65, 2%) developed a cerebrospinal fluid leak, which required OR takeback and operative intervention while under neurosurgical care. Some patients experienced more than one postoperative complication.

**Figure 3:**
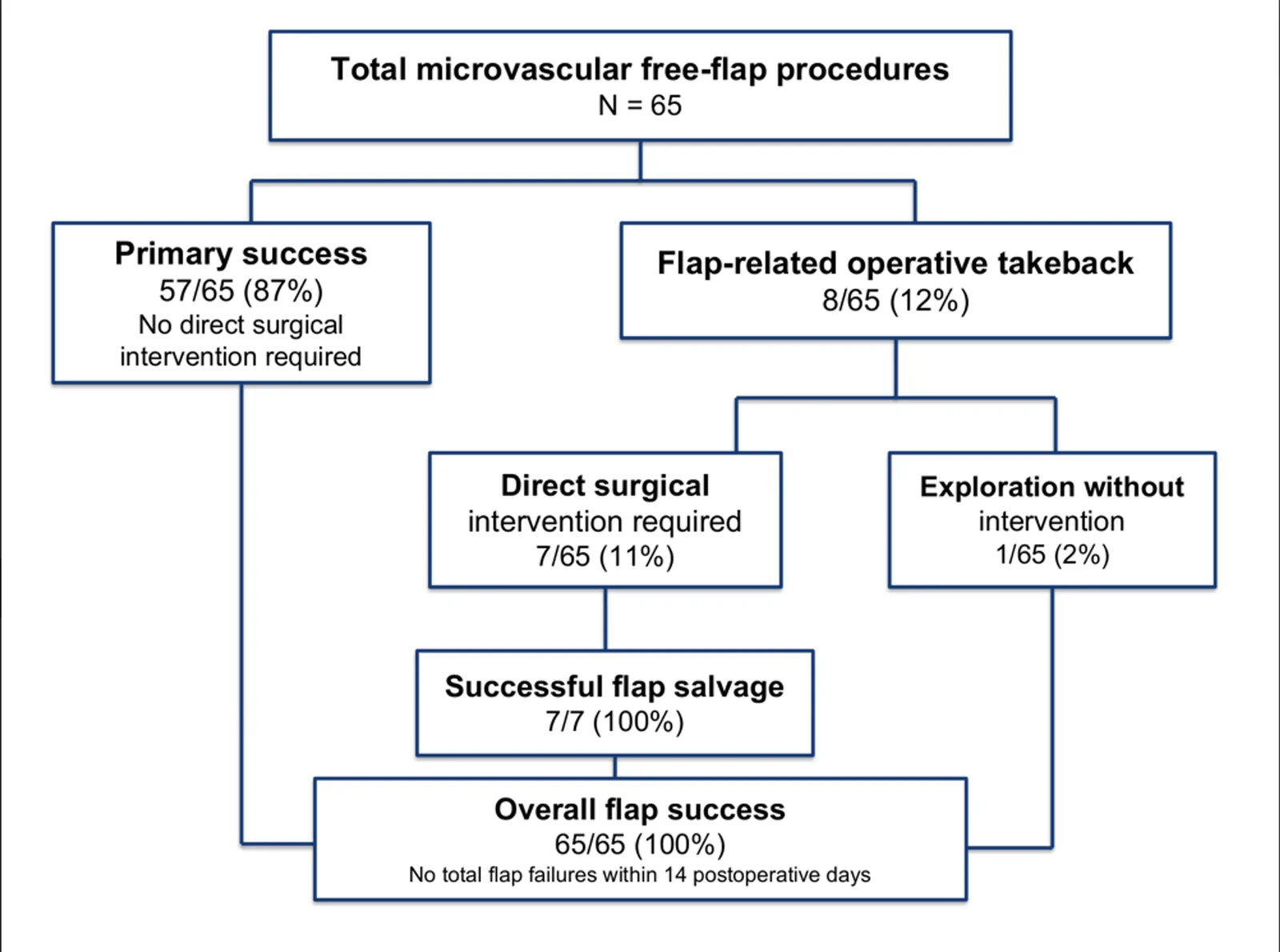
Free-Flap Success, Operative Takeback, and Salvage Outcomes Flow diagram of outcomes among 65 microvascular free-flap procedures. Primary success without direct surgical intervention occurred in 57/65 cases (87%). Eight patients underwent flap-related operative takeback (8/65, 12%); seven required direct surgical intervention (7/65, 11%), while one underwent exploration without intervention (1/65, 2%). All seven flaps requiring intervention were successfully salvaged, yielding a salvage rate of 7/7 (100%) and an overall flap success rate of 65/65 (100%). No total flap failures occurred within 14 postoperative days. The figure was created by the authors using Microsoft PowerPoint (Microsoft Corp., Redmond, WA, USA).

Primary success was defined as flap survival without direct surgical intervention. A total of 57/65 (87%) of the patients required no surgical interventions, whereas 7/65 (11%) required surgical intervention to maintain pedicle viability (Figure 3). All seven flaps requiring intervention were successfully salvaged, yielding a salvage rate of 7/7 (100%). Overall flap success was 65/65 (100%), with no total flap failures within 14 postoperative days.

As described above, 8/65 patients (12%) who underwent microvascular free-flap surgery required flap-related operative takeback under the otolaryngology service (Figure 3). Seven required direct surgical intervention to maintain flap viability, while one underwent exploration without intervention. Separately, two patients required neurosurgical re-intervention for complications related to the neurosurgical component of their care. These events did not involve the otolaryngology team and were not classified as flap-related takebacks.

Among the otolaryngology-related takebacks, seven (7/65, 11%) resulted in head and neck surgical salvage and one (1/65, 2%) in surgical exploration alone. Reasons for otolaryngology takeback included venous thrombosis (3/65, 5%), hematoma evacuation (2/65, 3%), removal of adhesions (1/65, 2%), Doppler wire replacement (1/65, 2%), and otolaryngologic surgical exploration alone (1/65, 2%).

The patient requiring neurosurgical re-intervention underwent an initial collaborative procedure involving both neurosurgery and head and neck surgery. The subsequent takeback was performed by the neurosurgical team for management of increased cerebrospinal fluid pressure related to the neurosurgical component of the operation and did not involve the head and neck surgeons.

## Discussion

The institutional free-flap failure rate was 0%, compared with a mean failure rate of 3.33% across the six selected high-volume comparator studies [2-4,11,13,14]. The institutional rate was significantly lower on Welch’s t-test. However, direct comparison should be interpreted cautiously as the comparator studies differed in sample size, patient population, reconstructive site, follow-up duration, and definitions of flap failure. Despite these differences, the present findings compare favorably with outcomes reported by high-volume centers and suggest that successful microvascular reconstruction can be maintained in a lower-volume regional setting.

The current findings are consistent with the generally favorable outcomes reported in high-volume microvascular reconstruction series. Crawley et al. reported a complete flap failure rate of 4.8% among 892 patients, while Corbitt et al. reported a failure rate of 1.3% in a cohort of 3,090 patients [2,3]. Las et al. similarly reported an overall success rate of 96% among 1,530 free-flap reconstructions [4]. The remaining selected comparator studies also reported low failure rates [11,13,14]. As previously noted, while direct equivalence is limited, it provides useful context for interpreting the absence of complete flap failure in the current cohort.

Additionally, we sought to characterize preoperative comorbidities, intraoperative factors, and postoperative complications within the study cohort. This is quite pertinent given Newfoundland and Labrador’s high morbidity rate [15]. Given the absence of total free-flap failures, formal risk-factor analysis was not possible, and these variables were summarized descriptively. Nevertheless, taken together, the findings of this study support the continuation of a microvascular surgery program within the province of Newfoundland and Labrador. Our findings are in line with past studies that have supported the use of low-volume microvascular surgery programs by reporting favorable outcomes in low-volume institutions [6,7]. To our knowledge, comparable data from a low-volume Canadian center have not previously been reported.

The complications noted in this study are consistent with those in the relevant literature, such as venous thrombosis, which is reported to be one of the most common reasons for free-flap failure [6,7]. It is also important to consider the high success rate of our institution in surgical takebacks. Published series from both high- and low-volume institutions have reported variable salvage rates following operative takeback, frequently exceeding 60% [2,3,5]. The 100% (7/7) salvage rate therefore provides additional context beyond the overall failure rate and may reflect effective postoperative monitoring, timely intervention, and accumulated microsurgical experience.

It is also important to consider the patient population itself when generalizing research findings. Currently, Newfoundland and Labrador has some of the highest rates of chronic illnesses in Canada. It has been noted that this province has the highest rate of diabetes, at 19% [16]. Newfoundland and Labrador also has the highest rates of heart disease, stroke, and hypertension in Canada, which have only been projected to increase [17,18]. Currently, 36% of the provincial population lives with at least one comorbid condition that can be influenced by modifiable risk factors [19]. Given the high rate of comorbidities in the general provincial population, it can be concluded that the population inherently presents an increased surgical risk and greater likelihood of adverse surgical outcomes. Given the favourable outcomes observed in this low-volume setting, prior high-volume fellowship training may represent one contributing factor; however, the present study was not designed to isolate the independent effect of fellowship training from other surgeon-, patient-, and institution-level factors.

Overall, these findings support the continued availability of specialized microvascular reconstruction within Newfoundland and Labrador. Maintaining a regional program may preserve access to timely care and reduce the need for patients to travel outside the province. The favorable outcomes observed in this cohort suggest that low institutional volume does not necessarily preclude successful free-flap reconstruction when care is delivered within an experienced, appropriately supported program.

Limitations

Despite the encouraging study findings, some limitations should be noted. Given the retrospective study design, data collection was based on medical records that may be subject to incomplete or inaccurate documentation. There were multiple cases in which ischemia time could not be determined. The volume of blood loss may also have been inaccurate because there is no institutional process to ensure exact measurement, as blood loss may not account for blood contained in surgical sponges or lost outside of measured collection systems.

Another limitation was that this analysis only included outcomes identified during the patient’s hospital admission and did not account for long-term follow-up after discharge. Therefore, no conclusions were drawn regarding longer-term flap outcomes or patient recovery following discharge.

A further limitation was the lack of available Canadian institutional data for comparison. As a result, the comparator failure rates used in the statistical analysis were taken from studies conducted outside Canada. These studies differed in sample size, patient population, reconstructive site, follow-up period, and definition of flap failure. Therefore, the comparison with these studies should be viewed as providing context for the institutional findings rather than as a direct comparison of effectiveness.

The statistical comparison also used six annual institutional failure rates and six published study-level failure rates. These observations were not weighted according to the number of procedures represented in each institutional year or comparator study. Given the small number of observations, the statistical findings should therefore be interpreted cautiously.

Although all seven flaps that required surgical intervention were successfully salvaged, the 100% salvage rate was based on a small number of events and should also be interpreted with caution. Outcomes were analyzed at the institutional level and were not separated by individual surgeon, and therefore inter-surgeon variability could not be assessed.

Finally, while both surgeons had completed high-volume microvascular fellowship training, this study did not include a comparison group of surgeons without similar fellowship training. Therefore, the study cannot determine whether fellowship training itself was responsible for the favorable outcomes observed. Other surgeon-, patient-, and institutional-level factors may also have contributed to the outcomes.

Implications for future practice and research

While this study compared surgical failure rates to high-volume institutions, it did not compare surgical takeback rates, as they have not been widely reported in the literature. Evaluating outcomes following operative takeback in addition to overall failure rates may provide further insight into the ability to recognize flap compromise and successfully salvage threatened flaps.

The outcomes of this study are conducive to positive impacts within medical practice. As previously noted, many professional organizations base their guidelines on high-volume statistics that often state that to maintain surgical competency, a surgeon must complete 20 cases per year [1]. The findings of this study add to the growing body of evidence favouring the maintenance of low-volume microvascular surgical programs. These findings support the hypothesis that prior high-volume fellowship training may contribute to favourable outcomes in low-volume settings, although this association was not directly tested in the present study. Moreover, this study supports the stance that, even at a low-volume surgical center, free-flap success can be better achieved in patients with multiple comorbidities when overseen by a surgeon with appropriate training experience.

## Conclusions

Given the rigidity outlined in the Canadian guidelines, it was imperative to determine whether the historical reasoning for this annual surgical quota held true for low-volume institutions in Eastern Canada. The results of our study challenge this paradigm, as our institution had no total flap failures within the 14-day post-operative period, with a 100% (7/7) successful salvage rate. This is significant in the context of the high rates of chronic disease morbidity in Newfoundland and Labrador. Our findings demonstrate that favourable microvascular free-flap outcomes can be achieved in a low-volume setting when care is provided by surgeons with high-volume fellowship training. While the present study was not designed to determine the independent effect of fellowship training, these findings suggest that low institutional volume does not necessarily preclude successful microvascular reconstruction within an experienced and appropriately supported program. Overall, our results support the continued maintenance of specialized microvascular reconstructive programs within the province while highlighting the need for further research examining the relationship between surgeon training, institutional volume, and outcomes.
